# Establishment of rat liver cancer cell lines with different metastatic potential

**DOI:** 10.1038/s41598-020-65338-w

**Published:** 2020-05-20

**Authors:** Lei Song, Jian-gang Zhang, Long Zheng, Xu Feng, Jie Hou, Huan-ling Zhang, Shu-feng Liu

**Affiliations:** 1 Department of Laboratory Animal Science of Hebei Medical University, Heibei Key Laboratory of Laboratory Animals, Shijiazhuang, 050017 Hebei, China; 2 Institute of Traditional Chinese Medicine, Tianjin University of Traditional Chinese Medicine, Tianjin State Key Laboratory of Modern Chinese Medicine, Tianjin, 300193 China; 3 Department of Pathology, The Third Hospital of Xingtai, Xingtai, 054000 Hebei, China

**Keywords:** Cancer models, Collective cell migration

## Abstract

The gloomy outcome of liver cancer is mainly due to the high rates of metastasis and recurrence, even after curative resection for early stage liver cancer. Our study was conducted to find the animal model suitable for the study of liver cancer metastasis. In our study, two liver cancer cells were obtained from N-nitrosodiethylamine (DEN) and N-nitrosomorpholine (NMOR) induced rats, and they were cultivated, screened and cloning cultivated. Bionomics of cells was analyzed. The results show that 2 cells had different metastatic potentiality. They were named Wrh-f2 and Wrh-s2, and they have the characteristics of Hepatocellular carcinoma cells. The bionomics of 2 cells showed: (1) The chromosome karyotype analysis showed that the mode of Wrh-f2 was 80–83 and Wrh-s2 was 55–57; (2) AFP positive cytoplasmic staining was observed in Wrh-f2 and Wrh-s2. Cytokeratin (CK) 7 and CK8 positive staining was present in Wrh-f2. CK8 positive staining was present in Wrh-s2; (3) The numbers of Wrh-f2 and Wrh-s2 that passed through the Transwells were 98 ± 12 and 55 ± 15;(4) Wrh-f2 had the significant higher colony formation (78%) than Wrh-s2(8%) (*P* < 0.01). (5) The animal models generated solid tumours when 2 cells were inoculated to nude mouse and rat. And Wrh-f2 developed stable pulmonary metastasis. The established cell lines with different metastatic potential showed obvious advantages over liver cancer in mimicking the biological properties of malignant liver cancer tumors. It provided a suitable model for the mechanism of liver cancer metastasis *in vivo* and *in vitro*.

## Introduction

Liver cancer is the fifth most common primary malignant tumor with increasing incidence^1^. Although many progesses in diagnosis and clinical treatment, and advances in surgical techniques have been achieved, the prognosis for the entire liver cancer population remains poor. To date, surgical resection has been accepted the best treatment for hepatocellular carcinoma^2^. However, a high incidence of recurrence and metastasis hinders the long-term prognosis of patients after surgical resection^3^. It has been more than a century since Paget proposed the seed and soil hypothesis to describe the mechanism by which cancer spreads or metastasizes throughout the body^4^. However, as one of the major malignancy in human, the molecular mechanism underlying its development is still undefined, partially due to a lack of an ideal animal model for liver cancer^5^. Therefore, a suitable liver cancer metastatic model system and cell lines with metastatic potential are needed for investigating the mechanisms, the development, recurrence, metastasis and testing of new therapeutic strategies^6^.

In previous studies, metastatic animal models and human liver cancer cell lines^7,8^ have been developed and well characterized to study the mechanism of liver cancer metastasis. Including the highly metastatic cell line MHCC97H and the low metastatic cell line MHCC97L^9^. Moreover, the liver cell with metastatic capability was significant for discovering liver cancer metastasis-related genes and proteins, and the interaction between the microenvironment and liver cancer invasion^10^. Actually, there still existed a lot of problems. One challenge is that the tumor tissue derived from patients and subsequently put into nude mice. But the tissue no longer behaves as it did in patients. Although it can still grow sometimes, it rarely metastasize either regionally or distantly^11^. They may change morphologically, biologically and biochemically when human tumor cells used in animals. To solve this problem, our research group decided to prepare cloned rat hepatocellular carcinoma cells, and then prepare rat transplanted hepatocellular carcinoma model for the study of hepatocellular carcinoma. We induced the rat liver cancer model. Liver tumors were harvested for primary culture of rat hepatoma cells. Two hepatocellular carcinoma cell lines were obtained by screening of cell penetration and clonal culture. These two cells have different metastatic potential, and the AFP (alpha fetoprotein) expression is positive, consistent with the characteristics of hepatocellular carcinoma.

Hepatocellular carcinoma metastasis is the most basic characteristic of cancer and the ultimate cause of most cancer deaths. By exploring the differences in the characteristics of cells with different metastatic potentials and successfully establishing a metastatic animal model, it will be beneficial to study the mechanism of liver cancer metastasis. Relevant hepatocellular carcinoma cell lines and animal models are helpful not only to explore the mechanism of occurrence, recurrence and metastasis of hepatocellular carcinoma, but also to evaluate the effectiveness of new therapies for hepatocellular carcinoma.

## Methods

### Agents

Medium DMEM was purchased from Corning. Fetal bovine serum (FBS) and pancreatin were purchased from GiBico. N-nitrosodiethylamine^12^ and N-nitrosomorpholine (NMOR) were purchased from Sigma (St, Louis, MO, USA). Matrigel was purchased from BD. Rabbit polyclonal antibody to AFP (14550-1-AP), Rabbit polyclonal antibody to CK7 (15539-1-AP), Rabbit polyclonal antibody to CK8 (10384-1-AP), Mouse monoclonal to beta actin (60008-1-lg) and all secondary antibodies were procured from proteinteck (Proteinteck, CHINA).

### Ethics statement

All animal studies were performed with the approval of the Laboratory Animal Ethical and Welfare Committee (AEWC) Hebei Medical University. In addition to, all methods were performed in accordance with the relevant guidelines and regulations of the Laboratory Animal Ethical and Welfare Committee (AEWC) Hebei Medical University.

### Preparation of hepatocellular carcinoma cell line

For all experiments and procedures, anesthesia was induced with 0.6% pentobarbital sodium (1 ml/100 g). Animals were sacrificed by taking off their necks.

Experiments were performed in normal SPF Wistar rats (120 ± 10 g). DEN (100 mg/kg body weight) were intraperitoneally injected into the Wistar rats. From the next day, 100 ppm NMOR supplemented water were provided to rats. Rats were measured every two weeks until clear liver tumor appears. There were 10 animals died prior to the experimental endpoint. The veterinarian diagnosed the cause of death as poisoning.

The sterile tumor tissues obtained at the end of 20th week were cut into pieces between 0.5 mm and 1.0 mm, then were pooled into culture flasks according to direct cell monolayer culture method and incubated at 37 °C in a humidified atmosphere containing 5%C0_2_−95% air. The cells reached 80% fusion and the passage ratio was 1:2.

### Sorting and cloning culture of cells

The cells were detached with trypsin and then suspended in DMEM and diluted to the appropriate concentration. In order to separate different metastatic potential cells, 1 × 10^5^ cells (in 200 μL DMEM) were added into the upper chamber of transwell coated with 150 μg Matrigel (BD Biosciences, MA, USA). After 24 hours’incubation at 37 °C, the cells passing through the membrane were collected as high transfer rate cells to cultivate in a bottle. The rest of cells continue to develop after 264 hours. The cells outstanding wear membrane were collected as low metastatic potential cells to cultivate continue.

For cell cloning culture, 1 cell per well were seeded on 96-well plates and cultured at 37 °C for 2 weeks. Single cell clones were obtained respectively.

### Bionomics of 2 cell lines with different metastatic potential

1. **Morphology**: Morphology of two cells were viewed under light microscope and transmission electron microscopy. Representative pictures were taken.
2. **Chromosomal karyotype analysis**: Two days after inoculation, the cells were cultured in medium containing 50 g/ml colchicine for 3 hours. Cells were treated with trypsin, incubated with 0.075 mol/L KCI for 15 min, and fixed with methanol/acetic acid (3:l). Slides were air-dried and stained with Giemsa. Karyotype analysis of their chromosomes was carried out.
3. **DNA analysis**: The cells were digested with trypsin and fixed with 75% ethanol. The cell cycle was analyzed by flow cytometry.
4. **Immunohistochemistry**: Cells cultured for 3 days were washed with PBS, fixed with cold methanol for 8–10 minutes, washed with PBS, then fixed with 4% paraformaldehyde for 15–20 minutes, and exposed to air to dry. Anti-AFP, anti-CK7 and anti-CK8 kits were used in immunohistochemistry. The dilution of anti-AFP and anti-CK7 was 1:50, The dilution of anti-CK8 was 1:100.
5. **Western blot and Realtime PCR** The total protein concentration of 2 cells was determined by BCA method (Beyotime, Shanghai, China). Two hundred micrograms of protein samples were resolved by SDS-PAGE (12%) and transferred to polyvinylidine difluoride membrane that was blocked in Tris-buffered saline (PBST) supplemented with 5% defatting milk powder and 0.05% Tween 20 for 2 h at room temperature. The blot was treated with AFP antibody overnight at 4 °C, washed three times in PBST (10 min each), and incubated with second antibody for 1 h at room temperature. The membrane was washed three times with PBST, Add the luminescent solution and figures were gained from chemiluminescence apparatus (Bio rad, USA). The dilution of anti-AFP and anti-β-actin was 1:2000. The dilution of second antibody was 1:5000.
  The mRNA was extracted from 2 cells and the level of Afp mRNA was analyzed by realtime PCR. Each specimen was repeated 3 times, with GAPDH as the corresponding internal reference.
6. **Cell migration**: 2 × 105 high and low metastatic cells were suspended in serum-free medium and the upper lumen was added. The medium containing 20% FBS was used as a chemical attractant in the lower chamber. The cells were cultured in a humidified incubator at 37 °C for 20 h. The cells migrating to the submembranous surface through the membrane pores were washed with PBS, fixed with 95% ethanol for 10 minutes, and stained with Giemsa. Take photos of the stained cells in each field, randomly select 6 fields, and count the number of stained cells in each room. Each experiment was in triplicate.
7. **Colony formation**: For colony formation assays,50 cells per well were seeded in 6-well plates and cultured at 37 °C for 14 days. At the end of the trial, colonies (at least 50 cells per colony) were counted directly on the plate. Each measurement shall be made in triplicate and each experiment shall be carried out at least three times.

### Tumorigenicity of 2 cells

For all experiments and procedures, anesthesia was induced with 0.6% pentobarbital sodium (1 ml/100 g). Animals were sacrificed by taking off their necks.

Experiments were performed in normal SPF Wistar rats (120 ± 10 g) and male nude mice (5 weeks old). There was no animal died because of illness prior to the experimental endpoint.

## Tumorigenicity in nude mice

### Five-week-old male nude mice were used

The mice were injected right subaxillary with 2 × 10^6^ cells and each cell line was injected to 5 mice. Tumor growth was observed every 1–2 d after injection.

4 × 10^6^ cells (0.04 ml) suspended in NS were injected into the mice under liver capsular. Concerning the number of mice per experiment, each group had 10 mice. Tumour size and metastasis were monitored every 7 d.

## Tumorigenicity in Wistar rats

### Twenty male Wistar rats weighing 120–130 g were used

Wistar rats were injected right subaxillary with 2 × 10^6^ cells and each cell line was injected to 5 rats. Tumor growth was observed every 1–2 d after injection.

4 × 10^6^ cells (0.04 ml) suspended in NS were injected into the rats under liver capsular. Concerning the number of rat per experiment, each group had 10 rats. Tumour size and metastasis were monitored every 7 d.

## Results

### Preparation of hepatocellular carcinoma cell line

**Figure 1:**
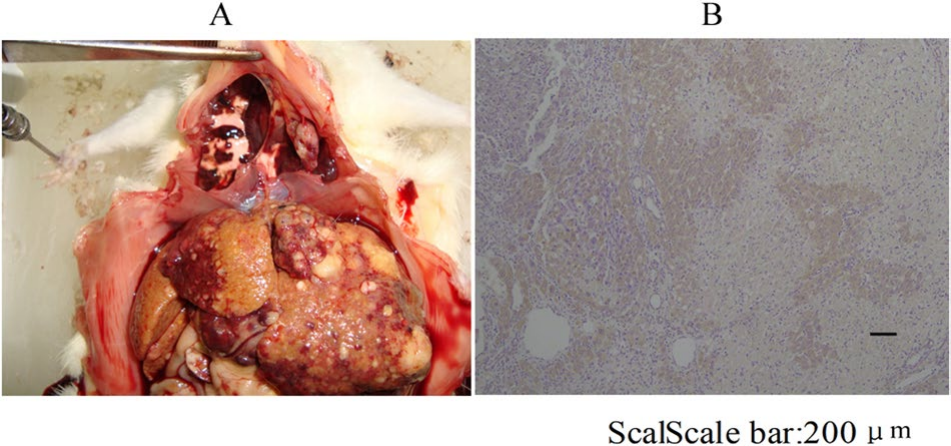
A rat model of hepatocellular carcinoma was reproduced at the end of 20 th week. (**A**) The anatomy of a liver tumor at the end of 20th week; (**B**) Immunohistochemical results of AFP in liver tumor tissue at the end of 20th week (100×).

Large nodules were appeared in the liver of induced liver cancer rat model at the 20 weekend, and 60% of pulmonary metastasis (Fig. 1A) . The results of modeling HE in liver and lung at different time points were shown in the previous literature published by the research group^13^. At 20 weeks, the liver cells presented different degrees of atypia, some of which showed tumor giant cells, little cytoplasm, poor differentiation, hemorrhage, necrosis and inflammatory cell infiltration, which were signs of advanced liver cancer. Immunohistochemical results of AFP in liver tumor showed strong positive expression of AFP (Fig. 1B).

To establish a liver cancer culture, a primary culture was made using the tumour derived from induced animals. Tumor tissues of liver were removed and used for primary culture *in vitro*^14^. Cells were seen migrating from the explanted tissue within 5 days. Cells passage culture was executed when cells covered 80% of flasks and did passage again after 10 days. At last, we succeeded in maintaining the primary cultured cells.

### Sorting and cloning culture of cells

1 × 10^5^ cells (in 200 μL DMEM) were added into the upper chamber of transwell coated with 150 μg Matrigel (BD Biosciences, MA, USA). After incubation for 24 h, significant migration was found. The cells passing through the membrane were collected as high transfer rate cells and cultivate in a bottle. Collected the cell on chamber membranes, named low migratory ability cells after following 264 h culture.

In order to get a single liver cancer cell lines, 2 cells were cloning cultured. It showed that clone was seen after 7 days in 96-well plate. Wells with multiple clones were abandoned. At last,2 cell lines were obtained inclding 1 high metastatic potential and 1 low metastatic potential, named Wrh-f2^15^ and Wrh-s2.

## Bionomics of 2 cell lines with different metastatic potential

### Morphology

**Figure 2:**
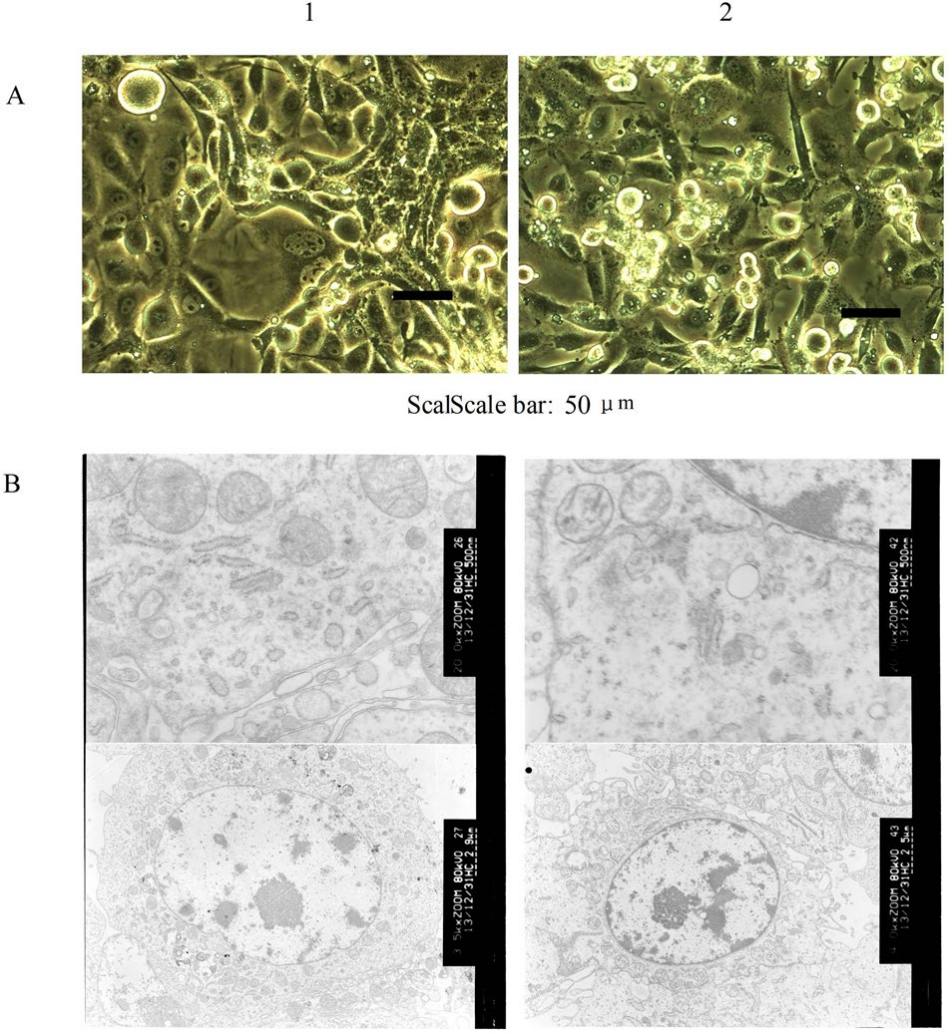
The Establishment of hepatocellular carcinoma cell lines (**A**) Microphotographs (400×) of the cultural cell lines. It has the same characteristics as the patient’s hepatocellular carcinoma tissue. Cells retained the features of hepatocyte-like cells such as the large nucleus and abnormal mitotic phase. (**B**) Electron microphotographs of cell lines displaying irregular nuclei, extension and particle fusion of rough endoplasmic reticulum and abundant microvilli on cell surface (above: 20000×; Bottom: 3500×) (1:Wrh-f2; 2:Wrh-s2).

Inverted phase contrast microscope showed 97% polygonal cells and 3% round cells. No desmocyte was observed. The cultured cells had large conspicuous nucleus, with coenocyte and abnormal mitotic phase, which were same with liver cancer tissue of paitient (Fig. 2A). The cells presented clone-like growth at low density wherease presented overlapping growth at high density with small tissue block structure seen by naked eyes and ambiguous boundaries, which implied the disappearance of contact inhibition^16^. Electron microscopy showed cells with magnocellular nucleus, irregular shape and mild deformity (Fig. 2B). Cells with multiply obvious nucleolus, extension of rough endoplasmic reticulum, particle fusion and degranulation were also observed. The number of free ribosomes decreased. Most cristae and fewer membrane of mitochondria hazily fused. Some rough endoplasmic reticulum emerged particle fusion and degranulation. Mitochondrions changed to vacuole^17^ Microvilli and mitochondria with myeloid metaplasia were observed on the surface of Wrh-f2 and Wrh-s2.

### Chromosomal karyotype analysis

**Figure 3:**
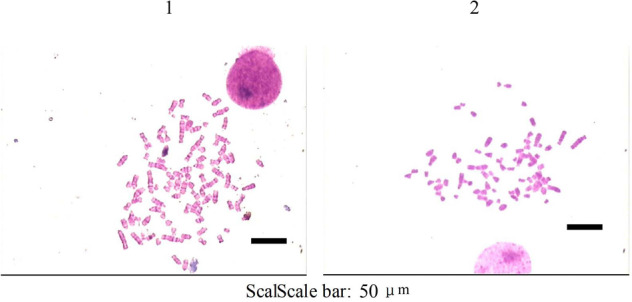
Karyotypes of 2 cell lines. Most of Wrh-f2 was hypertetraploid. Most of Wrh-s2 was hyperdiploid (400×) (1: Wrh-f2; 2: Wrh-s2).

Wrh-s2 were hyperdiploid and chromosome number was 23–64, mode was 55–57; Most of Wrh-f2 were hypertetraploid and chromosome number was 71–90, mode was 80–83. Internal diversity of cells varied greatly (Fig. 3).

### DNA analysis

**Figure 4:**
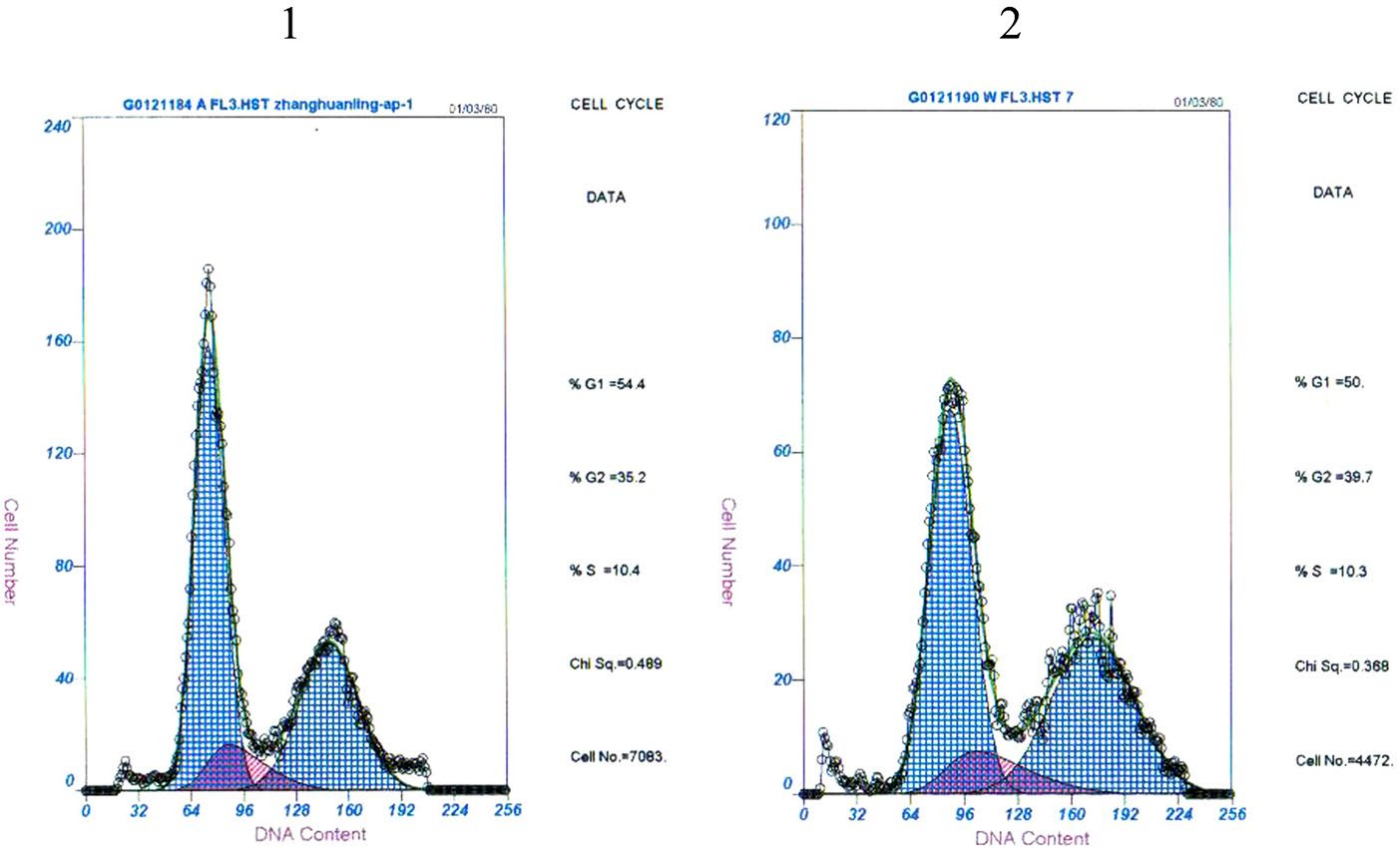
Distribution of DNA content detected by FCM. There were no significant differences in 2 cells (1: Wrh-f2;2: Wrh-s2).

In view of above mentioned characters, we were interested in researching whether the cell cycles were same or not, so cell cycle analysis were detected by flow cytometer. It was observed that there were no significant differences, and it was the proportion of cells in the G1 phase that occupies a leading position (Fig. 4).

### Immunohistochemtisty

**Figure 5:**
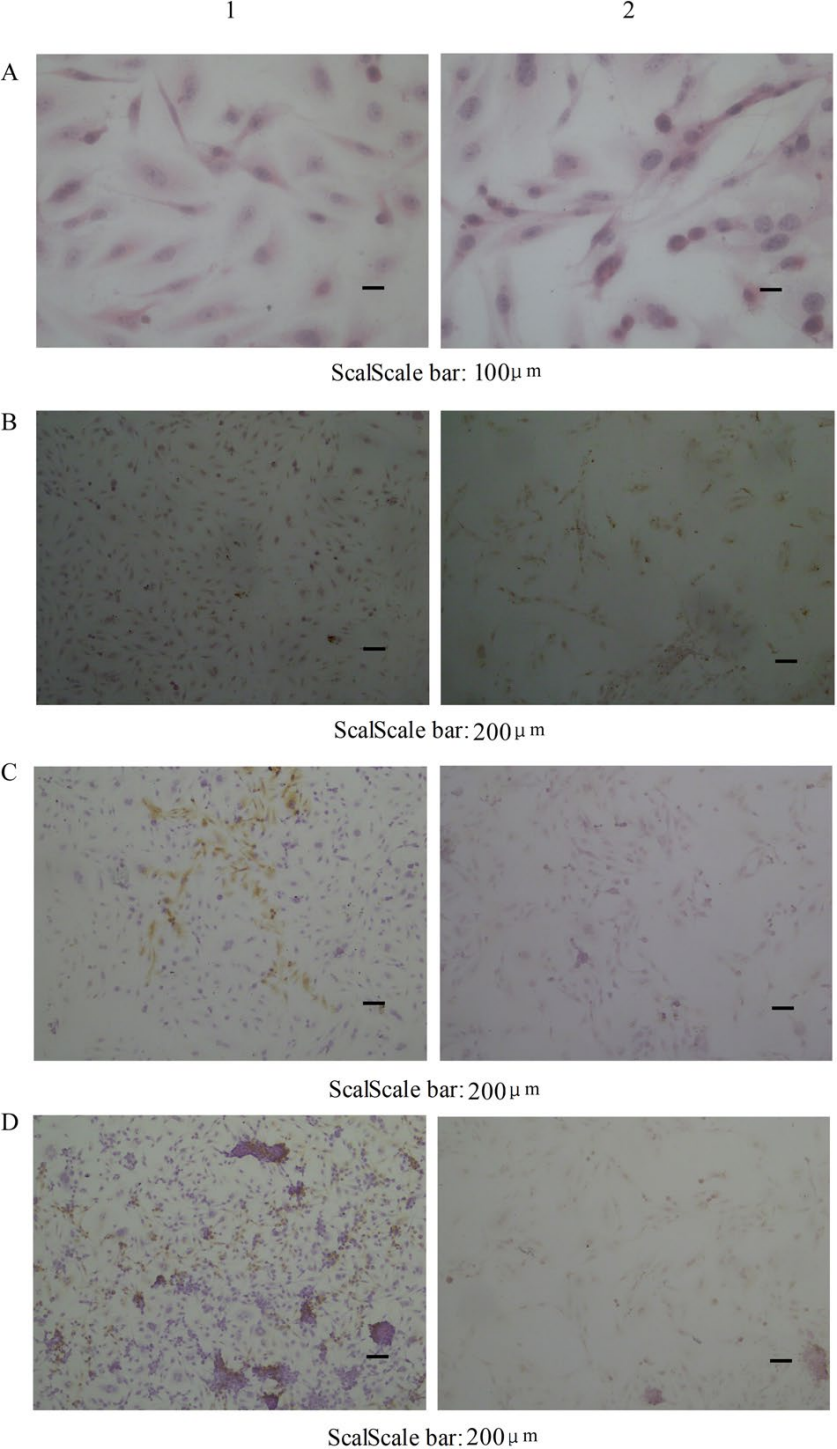
The micrographs of HE and immunohistochemical staining. (**A**) Microphotograph of HE staining (400×); (**B–D**) The micrographs of immunohistochemical staining (100×). Primary antibodies used are as follows:AFP (**B**), CK7 (**C**), CK8 (**D**) antibodies.(1: Wrh-f2; 2: Wrh-s2).

The expression levels of AFP and keratin were measured by immunohistochemical staining. Immunohistochemical analysis of AFP and keratin in hepatocellular carcinoma were revealed in Fig. 5A–D. AFP positive cytoplasmic staining was observed in Wrh-f2 and Wrh-s2. CK7 and CK8 positive staining was present in Wrh-f2^18^. CK8 positive staining was present in Wrh-s2.

### Western blot analysis

**Figure 6:**
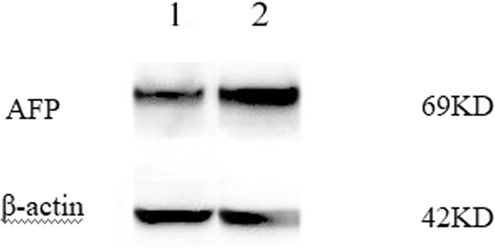
Expression levels of AFP in 2 cells were detected by Western blot analysis (The exposure time is 40 seconds) (1: Wrh-f2;2: Wrh-s2).

We then performed western blot analysis with anti-AFP to determine the amount of AFP in both cells. The spots at 69 kDa was confirmed as AFP. It was found that the expression of AFP in both cells was positive (Fig. 6).

### Realtime PCR

**Figure 7:**
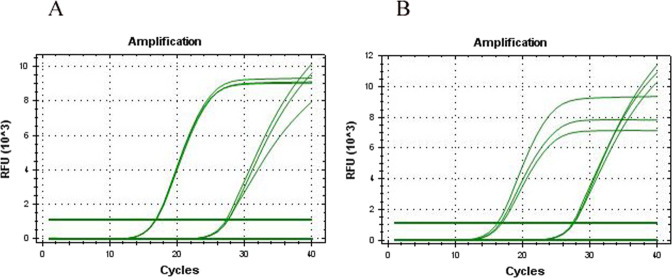
Levels of AFP mRNA in 2 cells were detected by Realtime PCR. (**A**) Wrh-f2; (**B**) Wrh-s2).

We performed Realtime PCR to determine the level of AFP mRNA in both cells. The results showed that the CT values of AFP amplification in the two cell lines were about 27.5, and the CT value of internal reference GAPDH was about 16.7. It was said that the level of AFP in both cells was positive (Fig. 7).

### Migration assay

**Figure 8:**
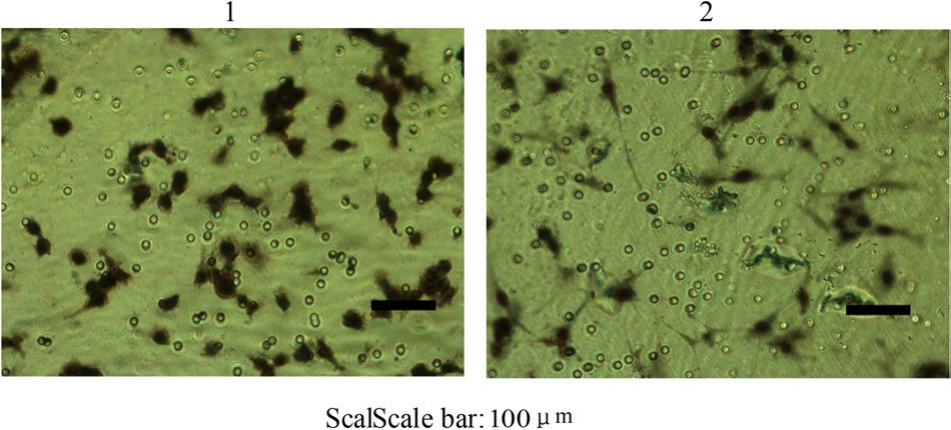
Migration potential was measured by transwell cell culture chamber (200×) (1: Wrh-f2; 2: Wrh-s2).

The cells that migrated to the basal side of the membrane were visualized and photographed with a microscope (Nikon, Japan) at 200 magnification. Images of 6 random fields from three replicate wells were obtained, and the cells that migrated were counted (Fig. 8)^19^. Numbers of cells named Wrh-f2 and Wrh-s2 that migrated were 98 ± 12 and 55 ± 15. They had extremely significant differences (*P* < 0.01).

### Colony formation

**Figure 9:**
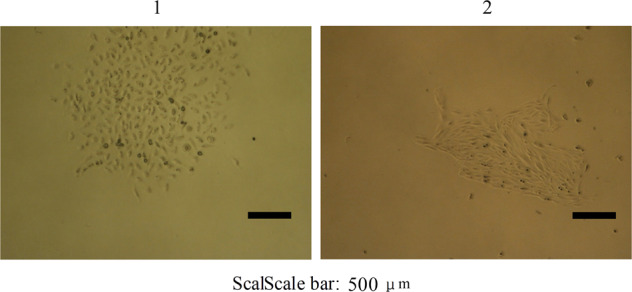
Colony formation assays. Representative images are shown (40×) (1: Wrh-f2; 2: Wrh-s2).

We counted and imaged the colony formation (Fig. 9). The highest was Wrh-f2 (78%), and the lowest was Wrh-s2 (8%). They had extremely significant differences (*P* < 0.01).

## Animal models

### Subcutaneous vaccination in nude mice

**Figure 10:**
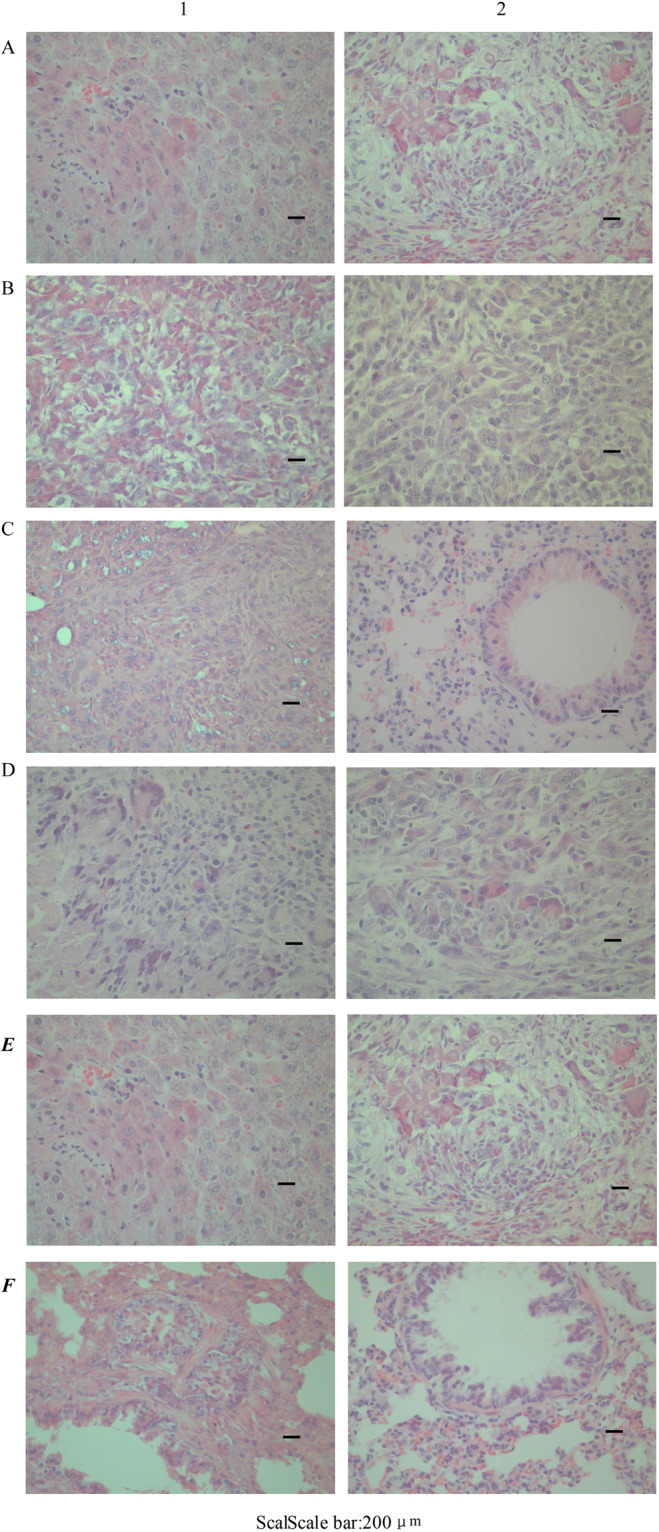
HE staining of tumors (100×). (**A**) Tumours derived from oxter of the nude mice; (**B**) Tumours derived from hepatic subcapsule of the nude mice; (**C**) Pulmonary metastasis occurred obviously in the nude mice which get vaccinated Wrh-f2 in hepatic subcapsular; (**D**) Tumours derived from oxter of rat; (**E**) Tumours derived from hepatic subcapsule of rat; (**F**) Pulmonary metastasis occurred obviously in rat which get vaccinated Wrh-f2 in hepatic subcapsular. (1: Wrh-f2; 2: Wrh-s2).

Male 5-week-old nude mice were injected subcutaneously with tumor cells. About 7 days later, the tumer volumes were measured. Tumor volume was calculated using the formula: (a × b^2^)/2, where a stands for the long diameter and b is the short diameter^20^. The tumor volumes of Wrh-f2 and Wrh-s2 reached 0.051 cm^3^ and 5.32 cm^3^. HE staining showed polygonal cancer cells with abundant cytoplasm, round or oval nuclei, obvious nucleoli, obvious atypia, and more mitotic phases^21,22^. Tumor giant cells were occasionally seen, which were arranged in strips with fibrous septum and rich interstitial blood vessels (Fig. 10A), which were consistent with human liver cancer. The results showed that the model was successfully constructed, and both cells had tumorigenicity.

### Hepatic subcapsular vaccination in nude mice

Two weeks after subcapsular inoculation of 2 HCC cells in nude mice, tumor growth was observed in the liver of both groups, and obvious lung metastasis was observed in group wrh-f2^23^. The incidence of pulmonary metastasis was 100%. HE staining of tumours was accordance with subcutaneous vaccination in nude mice (Fig. 10B). The lung HE staining of group wrh-f2 showed obvious infiltration of liver cancer cells (Fig. 10C).

### Subcutaneous vaccination in rats

About 14 days later, the tumer volumes were measured. The tumor volumes of Wrh-f2 and Wrh-s2 reached 0.52 cm^3^ and 7.68 cm^3^. The HE staining of tumours was accordance with Subcutaneous vaccination in rats (Fig. 10D). The results showed that the model was successfully constructed, and both cells had tumorigenicity.

### Hepatic subcapsular vaccination in rats

Liver tumor appeared after 2 weeks injection. The incidence and extent of lung metastasis were significantly different between Wrh-f2 and Wrh-s2. Lung metastasis rate for Wrh-s2 were 10%, whereas the rate for Wrh-f2 reached 80%. Compared with Wrh-s2, Wrh-f2 had a faster tumor growth and deeper pulmonary metastasis (Fig. 10E,F). The results showed that the model was successfully constructed, and both cells had tumorigenicity.

## Discussion

Tumor metastasis is a complex process, which includes a sequence of many steps, includes invasion, transport, arrest, adherence, extravasation, tumor cell proliferation and *et al*^24^.. To explore the mechanisms underlying metastasis, suitable models for cancer metastasis are necessary. A number of human liver cancer cells and animal models have been established^25^. These models have provided very beneficial tools for analysis of steps and individual treatment for tumour^26,27^. However, in order to analyze the natural procedure of tumour metastasis, it is essential to establish animal models and cell lines which can transfer stablely from primary tumors to other organs.

DEN and NMOR have been widely used as hepatocarcinogens in animal models. Futakuchi^28^ previously found that DEN exposure followed by a 16-week treatment with NMOR is a most efficient method for the induction of liver cancer metastasizing to the lung. Yoshino^29^ *et al*. modifed the experimental protocol and confirmed that lower doses of NMOR (40 and 80 ppm in drinking water) after DEN treatment significantly improved survival rate compared with the previous data with 120 ppm NMOR, and induced liver cancer with frequent metastatic potency to the lung. In our study, we use DEN (100 mg/kg) and NMOR (100 ppm) to induce carcinogenesis. Positive of AFP was detected immunohistochemically, and which appeared in an early stage of carcinogenesis^15^. The result reflected the malignant conversion of primary lesions. Furthermore, liver cancer with 60% lung metastasis was produced at 20-week. HE analysis of lung tissues confirms that lung metastasis really occurs with poor differentiation and inflammatory cell infiltration. We have successfully established a rat model of liver cancer, induced by DEN and NMOR, which showed obvious natural characters and lung metastasis. From this model, 2 different metastatic cell lines, Wrh-f2 and Wrh-s2 were screened and characterized. The 2 cells demonstrated remarkable difference in pulmonary metastasis after orthotopic inoculation. The differences in metastatic potential of Wrh-f2, and Wrh-s2 make them good materials for the research of molecular mechanisms and courses in liver tumour metastasis^30^. Becauce the 2 tumour cell lines originated from one rat model, they have the same genetic background. Consequently, comparisons of gene or protein expressions from 2 cells with different metastatic potentials could help to discover metastasis-related genes or proteins^31^. There are some similar reports at present, for example, expression of matrix metallo preteinases (MMP) is associated with high metastatic potential^32^.

Morphological and biochemical consequences can indicate hepatocyte origin as the source of cell lines^2^. The cultured cells have large conspicuous nucleus, which were same with liver cancer tissue of clinical paitients. The cells presented clone-like growth at low density wherease presented overlapping growth at high density with small tissue block structure seen by naked eyes, which implied the disappearance of contact inhibition. Cell cycle regulation is a crucial mechanism of cancers. There were no significant differences in cell cycle between the 2 cell lines. Cells in the G1 phase occupy a leading position. The result is accordance with Gerard^33^. As for chromosomal karyotype analysis, Wrh-s2 were hyperdiploid and most of Wrh-f2 were hypertetraploid. The theory, “diploid-polyploid conversion”, plays a vital role in physiopathological processes. Progressive polyploidization of hepatocytes takes place in liver tissue. The liver proliferate and adjust its ploidy when suffering liver stresses and injury signals (excess metabolism, toxic injury, chronic hepatitis etc)^34,35^. Unplanned genome-wide duplications induce polyploid cells. They can break the stability of chromosomes thus inducing carcinogenesis. Tetraploid cells have been detected in early stage of oncogenesis^36,37^. Expression of specific genes can be altered by polyploidy^38^. Indeed, in some tumor types, there is direct evidence for the development of aneuploidy from a transient 4n state^39^. But the impact of polyploid hepatocytes status on hepatocarcinoma (liver cancer) is still not exactly.

Alpha fetoprotein (AFP) expresses in embryonic tissues and silences in adult liver. In majority of liver cancer patients, the expression of AFP is reactivated, therefore, AFP has been generally accepted as a diagnostic and prognostic biomarker for liver cancer since 1970s^38,39^. The sensitivities and specificities of AFP were 41–65% and 80–94% respectively at any stage of liver cancer^40,41^. AFP is not only potential in early detection but allso is useful for liver cancer diagnosis as a general serum marker. Guidelines from American Association for the Study of Liver Diseases (AASLD) stated that the level of AFP exceeding 200 ng/mL was enough for tumor diagnosis^25^. In our study, AFP was tested by immunohistochemtisty, western blot assay and realtime PCR. Wrh-f2 and Wrh-s2 show positive AFP at the same time. Because the two cells had the same origin and were cloned, the cells were of a single type. Positive AFP proved that both cells were hepatocellular carcinoma cells. Interactions between tumor cells and extracellular matrix (ECM) are significant to invasion and metastasis^42^. Invasion and migration show significant differences, implying respective metastatic characteristics. Thus, the results above suggest that the natural properties and courses of obtained cell lines closely resemble those of the typical liver cancer patients^43^. Cytokeratin 7 (CK7) has been identified as a progenitor-cell marker in a rat liver cancer model^44^. In normal human liver, hepatocytes express CK8 and do not exhibit CK7^45^. Immunohistochemical study using CK7 might be a useful tool for evaluating liver cell regeneration, and histological examination of the states of liver regeneration might be valuable for the diagnosis and evaluation of treatment response^46^.

A method called “orthotopic implantation” has been applied to developing animal models of liver carcinoma^47^. The lung is commonly affected by extrahepatic metastasis in liver cancer^48^. Liver cancer patients with distant metastasis have a dismal outcome and are universally incurable^49^. We inoculated tumour cells to nude mice and rats. In nude mice, liver appeared nodules and larger tumor tissues, what’s more, pulmonary metastasis rates were up to 80–100% in group Wrh-f2, which mimics liver cancer in person well. In rats, tumor volumes can be measured after 7 days and pulmonary metastasis rates dramatically differ. The incidence and extent of lung metastasis were significantly different between Wrh-f2 and Wrh-s2. Lung metastasis rate for Wrh-s2 were 10%, whereas the rate for Wrh-f2 reached 80%. Compared with Wrh-s2, Wrh-f2 had a faster tumor growth and deeper pulmonary metastasis. This model substantially enhances lung metastasis of liver cancer^50^ and can facilitate comparative study of the mechanism of tumor metastasis.

In summary, we have recently established 2 cell lines (Wrh-f2 and Wrh-s2). And they have different metastatic potential. 2 cell lines inoculate rat can produce lung metastasis model. The established cell lines with heteroploid karyotype are poor differentiated and have obscure bounds^51^. They can shorten the incubation for growth and metastasis. The constructed model of hepatocellular carcinoma shows a variety of essential behaviours that appear in tumor patients. Thus, the model has obvious advantages in elucidation of the mechanisms of malignant tumor metastasis, in predicting drug response, in development of anti-metastatic agents and in the evaluation of the efficacy of therapeutic treatments against liver tumor *in vivo*^21^.

## Conclusions

2 cell lines (Wrh-f2 and Wrh-s2) were tumorigenic in both nude mice and rats, and the tumor showed AFP positive, which was consistent with the characteristics of hepatocellular carcinoma. Among them, wrh-f2 has a low degree of differentiation, a high degree of malignancy and a high metastasis potential, which can lead to typical pulmonary metastasis, while wrh-s2 is a poorly differentiated cell with a relatively low metastasis potential and a low incidence of pulmonary metastasis.

## Supplementary information

Supplementary Information File.

## Data Availability

The data used to support the findings of this study are available from the corresponding author upon request.
