# Supplementary material for: Establishment of rat liver cancer cell lines with different metastatic potential
Source: Sci Rep. 2020 May 20;10:8329. doi: 10.1038/s41598-020-65338-w (PMC7239898; doi:10.1038/s41598-020-65338-w)
Supplement: Supplementary file 1 — Supplementary Information File. [file 41598_2020_65338_MOESM1_ESM.pdf]

Supplementary Information File

Establishment of rat liver cancer cell lines with different metastatic potential

Lei SONG[1, 2], Jian-gang ZHANG[3], Long ZHENG[1],Xu FENG[1],Jie HOU[1],Huan-ling ZHANG [1*], Shu-feng LIU [1*]

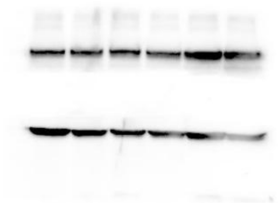

Fig.6 Original figure

(This is a picture of the six cell lines obtained in our experiment. The third column is the Wrh-f2 cell and the fifth column is Wrh-s2 cell.The top row is the band of afp, and the bottom row is the band of β-actin.The exposure time is 40 seconds)

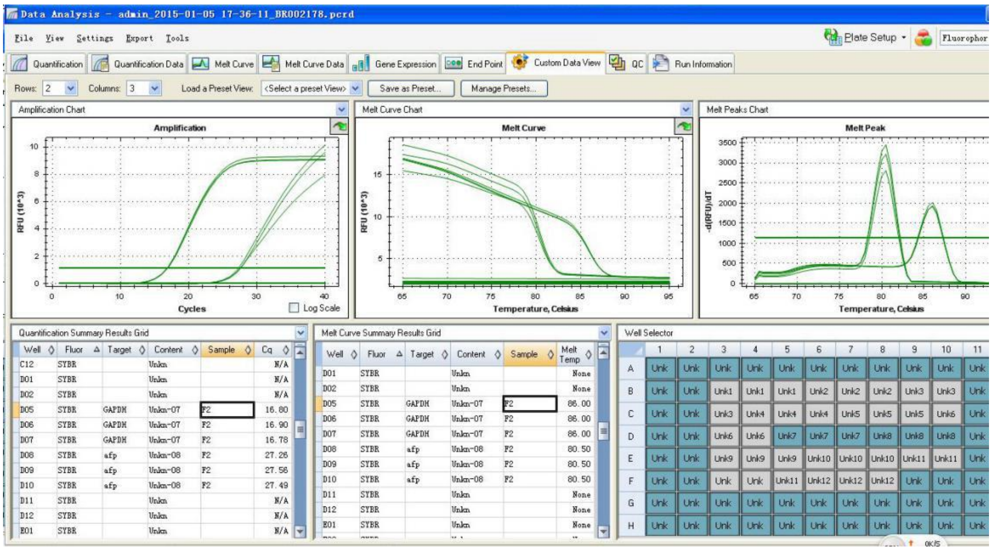

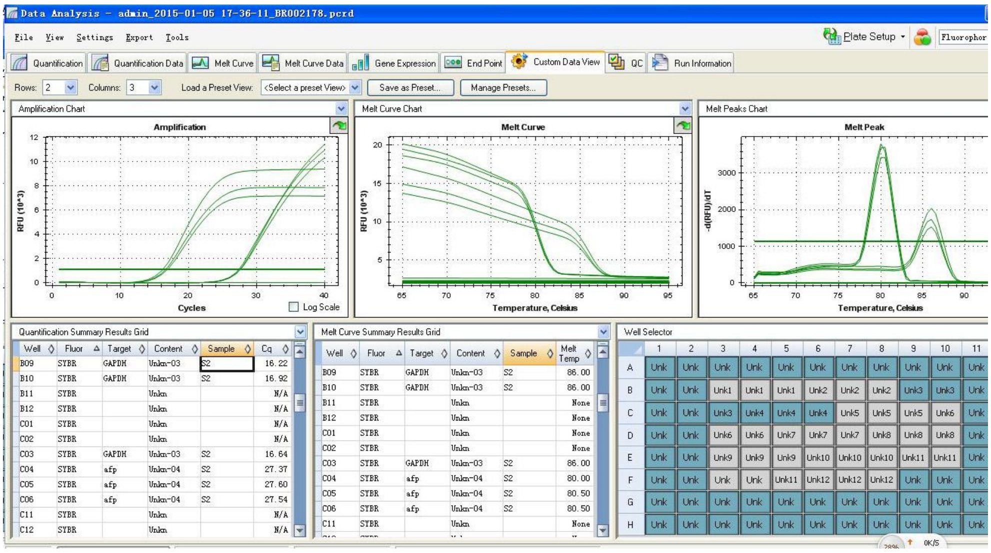

Fig.7 Original figure

(These two images are the realtime-PCR results of 2cell lines. The top image is Wrh-f2 cell , and the bottom image is Wrh-s2 cell.)
